# Diagnostic performance evaluation of post-operative serum calcitonin (CT) levels as a factor for good prognosis in medullary thyroid carcinoma (MTC)

**DOI:** 10.1186/1756-6614-6-S2-A17

**Published:** 2013-04-05

**Authors:** Danuta Gąsior-Perczak, Aldona Kowalska, Iwona Pałyga, Anna Słuszniak, Ryszard Mężyk, Stanisław Góźdź

**Affiliations:** 1 Holycross Cancer Centre, Kielce, Poland

## Introduction

The 10-year survival rate of MTC patients is 65%. Metastases may occur even many years after the surgery. Thus, MTC patients require constant oncological monitoring. The aim of the study was to assess the post-operative levels of basal and stimulated CT, as a prognostic factor for good prognosis in MTC patients.

## Patients

The study included 67 MTC patients (53 women and 14 men, aged 16-77 years) treated in our hospital (from 1996 to 2012).

## Methods

Basal and stimulated CT levels were measured 3 months after the surgery. The follow-up was continued for average 7.2 years (1-16 years). In the event of increasing CT levels, imaging studies were performed to detect metastases. Correlation between the occurrence or lack of metastases and CT levels 3 months after the surgery was assessed.

## Results

Metastases to cervical lymph nodes were found in 6 patients 1 to 10 years after the surgery, one patient had metastases to ovary 11 years after the surgery, one patient had metastases to liver 5 years after the surgery and 6 cases had a constant elevation of CT levels without any findings in imaging studies. 4 patients died 1-4 years after the surgery. Post-operative CT levels in all patients mentioned above were elevated (basal CT 20-518 pg/ml; stimulated CT 199-3823 pg/ml) In the rest of the patients with normal CT level no metastases or increase in CT levels were found during the follow-up (basal CT <2.0-12 pg/ml; stimulated <2.0-19.7 pg/ml).

## Conclusions

1. Normal basal and stimulated CT levels 3 months after the MTC surgery correlate very well with a good prognosis and lack of late metastases.

2. Further studies are required to confirm that patients with normal post-operative CT levels can be released from oncological monitoring.

